# *MLL* leukemia-associated rearrangements in peripheral blood lymphocytes from healthy individuals

**DOI:** 10.1590/S1415-47572009000200005

**Published:** 2009-06-01

**Authors:** María Sol Brassesco, Ana Paula Montaldi, Diana Ester Gras, Rosane Gomes de Paula Queiroz, Nilce Maria Martinez-Rossi, Luiz Gonzaga Tone, Elza Tiemi Sakamoto-Hojo

**Affiliations:** 1 Departamento de Genética, Faculdade de Medicina de Ribeirão Preto, Universidade de São Paulo, Ribeirão Preto, SPBrazil; 2 Departamento de Puericultura e Pediatria, Faculdade de Medicina de Ribeirão Preto, Universidade de São Paulo, Ribeirão Preto, SPBrazil; 3 Departamento de Biologia, Faculdade de Filosofia, Ciências e Letras, Universidade de São Paulo, Ribeirão Preto, SPBrazil

**Keywords:** genomic instability, lymphocytes, *MLL* rearrangements

## Abstract

Chromosomal translocations are characteristic of hematopoietic neoplasias and can lead to unregulated oncogene expression or the fusion of genes to yield novel functions. In recent years, different lymphoma/leukemia-associated rearrangements have been detected in healthy individuals. In this study, we used inverse PCR to screen peripheral lymphocytes from 100 healthy individuals for the presence of *MLL* (Mixed Lineage Leukemia) translocations. Forty-nine percent of the probands showed *MLL* rearrangements. Sequence analysis showed that these rearrangements were specific for *MLL* translocations that corresponded to t(4;11)(q21;q23) (66%) and t(9;11) (20%). However, RT-PCR failed to detect any expression of t(4;11)(q21;q23) in our population. We suggest that 11q23 rearrangements in peripheral lymphocytes from normal individuals may result from exposure to endogenous or exogenous DNA-damaging agents. In practical terms, the high susceptibility of the *MLL* gene to chemically-induced damage suggests that monitoring the aberrations associated with this gene in peripheral lymphocytes may be a sensitive assay for assessing genomic instability in individuals exposed to genotoxic stress.

## Introduction

Lymphoid neoplasias are generally characterized by the presence of chromosomal anomalies, the most prominent of which are those that produce in-frame fusion genes. These aberrations are important diagnostic tools that can be used to establish the prognosis of leukemias and lymphomas and monitor their progress.

Rearrangements of the *Mixed Lineage Leukemia* (*MLL*) gene generated by reciprocal translocations involving chromosome band 11q23 are well-known in infants and adults with acute myeloid leukemia (AML) and acute lymphoblastic leukemia (ALL), as well as in 85% of secondary leukemias associated with a history of treatment with topoisomerase II inhibitors (Adler *et al.*, 1999; Ross, 2000). More than 50 fusion genes involving *MLL* associated with a poor prognosis have been identified (Popovic and Zeleznick-Le, 2005; Slany, 2005). However, despite the diversity and frequency of *MLL* translocations, most of the breakpoints have been mapped within a *Bam*H1-delimited region known as the break cluster region or BCR (Sai-Peng and Liu, 2001) between exons 8 and 14 (Nilson *et al.*, 1996; Schnittger, 1998; Echlin-Bell *et al.*, 2003). Other aberrations involving *MLL* in leukemia include *in tandem* duplications represented by in-frame repetitions of exons 2-6 (or 2-8) that can be attributed to homologous recombination mediated by *Alu* repeats (Strout *et al.*, 1998; Whitman *et al.*, 2001).

Since the development of leukemia and solid tumors is a multistage process that requires multiple cooperative mutations, it seems plausible that different mutations, such as typically found in patients with leukemia or lymphoma, could arise in normal individuals (Hunger and Cleary, 1998).

The presence of tumor-associated fusion genes in healthy donors has been described for the translocation t(14;18) *IGH/BCL2* (characteristic of non-Hodgkin lymphomas), with variable frequencies (16.2%-55%) among populations and a tendency to increase with age (Liu *et al.*, 1994; Summers *et al.*, 2001; Yasukawa *et al.*, 2001). This rearrangement has also been described in 43% of blood samples from patients with non-proliferative malignancies (Rauzy *et al.*, 1998). Similarly, the translocation t(9;22) *BCR/ABL* was primarily detected in peripheral lymphocytes of adults and children (Biernaux *et al.*, 1995); Bose *et al.* (1998) subsequently confirmed these data by demonstrating p190 and p210 transcripts in 4 of 11 and 11 of 16 individuals, respectively. Other markers have also been detected at low frequencies in normal populations, including *ETV6/RUNX1* (Eguchi-Ishimae *et al.*, 2001; Brassesco *et al.*, 2004), t(11;14)(p13;q11) *LMO2/TCR* and t(7;14)(q34;q11) *TCR/TAL2* (Marculescu *et al.*, 2002) and t(15;17) *PML/RARA*, the latter characteristic of promyelocytic leukemia (Quina *et al.*, 2000). The incidence of *MLL* duplications in healthy donors is much higher, and are detectable in almost all samples by using sensitive PCR methods (Bäsecke *et al.*, 2006). Together, these studies indicate that leukemia and lymphoma-associated translocations can be generated in normal hematopoietic cells without apparent oncogenic consequences.

Based on these findings, we used an inverse-PCR strategy to investigate the presence of *MLL* translocations in peripheral blood lymphocytes from healthy individuals. Our results demonstrate the presence of *MLL* fusions in these cells, thus indicating that these rearrangements are not restricted to malignant cells but may also be present in a subset of normal hematopoietic cells.

**Figure 1:**
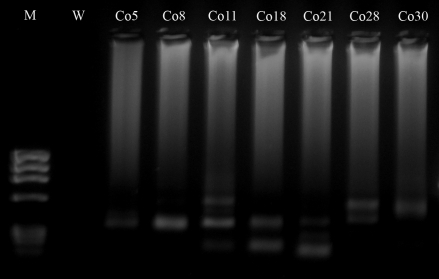
Representative agarose gel showing PCR amplimers that probably resulted from translocation events at 11q23 (*MLL*) in peripheral lymphocytes of healthy individuals. M = molecular weight marker ΦX 174; W = negative control; Co = control.

**Figure 2:**
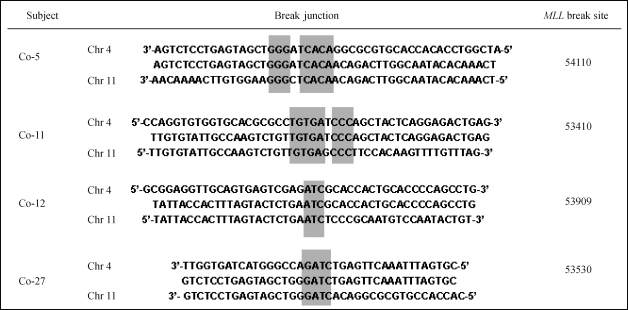
Illustrative *MLL* translocations detected by inverse PCR in four subjects. Individual breakpoints are flanked by the germ-line *MLL* sequence and sequences at chromosome 4. “Break site” refers to the position relative to the *MLL* gene (GenebankTM accession number Y373585) fused to its partner. Grey boxes indicate short microhomologies between *MLL* and *AF4* (4q21).

## Material and Methods

### Probands

Blood samples from 100 normal subjects (50 males, 50 females) were analyzed in this study. All of the subjects were healthy non-smokers 18 to 46 years old (mean ± SD = 22.9 ± 5.4 years) with no previous history of drug treatment or chronic use of medicines. A single sample of 10 mL of peripheral blood was obtained from each individual after informed consent, and the samples were immediately coded to ensure the anonymity of the donors. The study was approved by the local ethics committee of the Clinical Hospital of the Faculty of Medicine (University of São Paulo, Ribeirão Preto, SP).

### Translocation analysis by inverse-PCR

Inverse PCR was done according to Betti *et al.* (2001) with few modifications. Three micrograms of DNA was digested with a combination of *Sau*3AI and *Xba*I (10 units each) at 37 °C overnight. The addition of *Xba*I prevented amplification of the native *MLL* gene while allowing the amplification of translocation products that lacked the *Xba*I recognition site. After digestion, the samples were heat-inactivated at 65 °C for 10 min and then purified with a Wizard SV Gel and PCR Clean-up System Kit (Promega Corporation, Madison, WI, USA) to remove residual enzymatic activity. Following re-suspension in nuclease-free water, 0.5 μg of digested DNA was self-ligated in the presence of 3 units of T4 DNA ligase in a final volume of 20 μL for 16 h at 16 °C. All of the ligation reactions were terminated by incubation at 65 °C for 10 min. Eight microliters of ligated DNA was used in each PCR reaction. Nested primers were used to analyze the cleavage site at exon 12 of *MLL* in two 28-cycle reactions at temperatures of 95 °C, 55 °C and 72 °C for 1 min/step.

The following primers were used: *foward-1* 5'-CTT TGTTTATACCACTC-3'; *reverse-1* 5'-TAGGGAATAT AAAAGAGTGGG-3'; *forward-2* 5'-TTAGGTCACTTA GCATGTTCTG-3' and *reverse-2* 5'-CAGTTGTAAGGT CTGGTTTGTC-3'. Strict precautions were taken in each step to avoid cross-contamination of the samples.

### Analysis of translocation DNA sequences

PCR amplicons were separated on 1% agarose gels. Individual I-PCR products were extracted from the gels with a GFX PCR DNA and Gel Band Purification kit (Amersham Biosciences, Buckinhamgshire, UK). The fragments were then cloned into the pGEM-T cloning vector (Promega Corporation, Madison, WI, USA), transformed into pMOS Blue *Escherichia coli* and selected on LB-agar plates containing ampicillin (50 μg/mL), according to the manufacturer's instructions. Individual transformed colonies were then expanded for 22 h in liquid culture. Three hundred nanograms of plasmidial DNA was used as a template for the sequencing reaction with a Big Dye Terminator Cycle Sequence Ready Reaction kit (Amersham Biosciences) and the products were analyzed in an ABI Prism 377 DNA Sequencer (Applied Biosystems, Wellesley, MA, USA). Quality analysis and the removal of vector sequences were done with phredPhrap software (Ewing *et al.*, 1998; Ewing and Green, 1998). The resulting DNA sequences were then used to search the National Center for Biotechnology Information database with the Basic Local Alignment Search Tool (BLAST).

### Detection of *AF4/MLL* translocations by RT-PCR

Leukocytes from 22 healthy individuals were separated on Hystopaque-1077 (Sigma, St. Louis, MO, USA) and total RNA was extracted with TRIzol reagent (Gibco, BRL, USA), according to the manufacturer's instructions. After confirming the RNA quality by electrophoresis on a 1% agarose gel, reverse transcription was done using a High-Capacity cDNA Archive kit (Applied Biosystems, Foster City, CA, USA). Qualitative RT-PCR for the detection of translocation t(4;11)(q21;q23) was done according to the standardized protocol described in the BIOMED-1 Concerted Action Report (Van Dongen *et al.*, 1999) using the primer sets: *MLL-A* -CCGCCTCAGCCACCTAC-, *AF4-B* -TGTCACTGAGCTGAAGGTCG-, *MLL-C* -AG GACCGCCAAGAAAAGA-, *AF4-D* -CGTTCCTTGCT GAGAATTTG- and *MLL-E* -AAGCCCGTCGAGGAAA AG-.

### Lymphocyte culture

Lymphocytes were cultured using a standard protocol in which 0.5 mL of peripheral blood was added to 10 mL of RMPI 1640 medium (Sigma) supplemented with 20% fetal calf serum, 2% phytohemagglutinin (PHA) and penicillin/streptomycin. The cells were incubated at 37 °C for 72 h and treated with colchicine (0.56%) for the final 90 min. Cell harvesting and slide preparation were done using standard methods. Slides for FISH were stored at -20 °C until used.

### Fluorescence *in situ* hybridization

FISH was done using the commercially available probes LSI MLL Break Apart Rearrangement, according to the manufacturers protocol (Vysis, Downers Grove, IL). The probe labeled with SpectrumGreen covered a 350 kb portion centromeric to the *MLL* gene breakpoint region whereas the SpectrumOrange-labeled probe covered a 190 kb portion telomeric to the BCR. The expected signal pattern for a normal cell nucleus was two green(yellow)orange signals. In cells with *MLL* translocations, the green and orange signals were separated without the yellow intersection. The advantage of this strategy was that it allowed the detection of translocations regardless of the partner involved. At least 1000 nuclei were analyzed and images were captured with an Axiovision System (Zeiss, Germany).

## Results

Inverse PCR was used to screen the peripheral blood lymphocytes of normal individuals for *MLL* translocations. In this strategy, the translocation region was excised with restriction enzymes, circularized and amplified using various *MLL* primers. This approach allowed the detection of any rearrangement involving the cleavage site at exon 12, which contains putative topoisomerase II recognition sequences and is sensitive to DNAse I and some cytotoxic agents.

Forty-nine of the 100 DNA samples that were screened contained bands of variable sizes that corresponded to alterations spanning the *MLL* breakpoint region (Figure 1). In gel electrophoresis, the putative translocations resulted in one, two or three amplification products of 300-700 bp (amplification of the germ-line *MLL* was prevented by treatment with *Xba*I), which suggested that some individuals may have more than one *MLL* translocation. The individual bands were separated by electrophoresis in 1% agarose gels and cloned into the pGEM-T vector, transformed in *E. coli* pMOS Blue cells and sequenced. BLAST analysis of individual amplicons confirmed that these rearrangements were unique and specific for *MLL* rearrangements. Of the 35 clones that were obtained, 66% contained the translocation t(4;11)(q21;q23), which fuses *MLL* and *AF4* and occurs mainly in acute lymphoblastic leukemia. The remaining translocations fused *MLL* to sequences located on chromosomes 1, 2, 9 (7 cases), 12 (2 cases) and 19 (Table 1). Although these chromosomes contain known *MLL* partner genes, such as *EPS15* (1q32), *MLLT11* (1q21), *AF9* (9q34), *CIP29* (12q13), *ELL* (19q13) and *EEN* (19q13) (Atlas of Genetics and Cytogenetics in Oncology and Haematology), the partner sequences did not match with specific chromosome bands. Interestingly, sequence analysis of the breakpoint junctions revealed short microhomologies (1-8 bp) suggestive of non-homologous end joining repair (NHEJ) (Figure 2).

Chromosomal preparations from 49 individuals were also analyzed using the LSI MLL (Vysis) commercial probe, which allows the detection of different rearrangements at 11q23. At least 1000 nuclei were analyzed per individual and the translocation frequencies were found to vary from zero to 0.3 events/100 cells (mean ± SD = 0.04 ± 0.06). The specific probes also allowed the detection of extra signals, with frequencies ranging from zero to 0.79 signals/100 cells (mean ± SD = 0.18 ± 0.19) (Table 2).

Together, these results raised the question of whether *MLL* fusion genes were expressed at a transcriptional level. Since t(4;11) was the most frequent translocation, RNA samples from 22 donors were screened for *MLL/AF4* transcripts using different primer sets that allowed the detection of all known fusion transcripts between exon 8 of *MLL* and exon 7 of *AF4*. No t(4;11)(q21;q23) transcripts were detected by RT-PCR in peripheral lymphocytes from healthy individuals (data not shown).

## Discussion

The potential of the *MLL* gene for recombination makes it difficult to detect aberrations by classic methods. Consequently, the detection of *MLL* translocations is a challenge because although they have a known 5' sequence their 3' end can be one of a wide variety of translocation partners. The use of inverse PCR eliminates this problem by amplifying circularized fragments derived from any segment flanking a known DNA sequence. As shown here, *MLL* fusion genes were detected in the peripheral lymphocytes of 49 of the 100 normal individuals examined in this work. The presence of these rearrangements was confirmed by FISH on interphase nuclei, and showed that 28.5% of the samples showed signal separation (based on the use of a specific dual-color “split-signal” DNA probe). The discrepancy between the results obtained with these two methods probably reflects the difference in their sensitivities: whereas FISH can detect one positive cell in a thousand, PCR-based techniques can detect one cell in a million. Interestingly, 21 individuals who were negative for rearrangements by FISH had extra signals for the *MLL* gene by PCR; these extra signals probably represented translocations with other gene partners.

Direct DNA sequencing of the inverse PCR amplicons showed that most of the fusion sequences were t(4;11)(q21;q23); however, no *AF4/MLL* transcripts were detected by RT-PCR (standardized for the study of minimal residual disease) in 22 RNA samples.

The results of this study show that *MLL* rearrangements are not restricted to malignant cells but may also occur in normal hematopoietic cells. As indicated above, several studies have reported the presence of leukemia-lymphoma-associated fusion genes (*e.g.*, *BCR/ABL1, IGH/BCL2, TCR*β/γ) in normal individuals. *In tandem* partial duplications of *MLL* have been detected in almost all bone marrow and peripheral blood samples from healthy donors (Schnittger *et al.*, 1998; Bäsecke *et al.*, 2002, 2006), but there is only one report of translocations involving *MLL* in normal individuals. Uckum *et al.* (1998) used nested PCR to show that rearrangements involving *MLL* and the transcription factor *AF4*, resulted in the translocation t(4;11)(q21;q23) in bone marrow samples from fetuses and normal children, as well as in fetal liver samples.

These findings indicate that such translocations *per se* do not define clinically apparent diseases, but rather that malignant progression appears to depend on additional factors such as the occurrence of oncogenic secondary alterations. Leukemia-associated gene fusions are generally believed to occur *in utero*, before birth. For twins with concordant leukemia and *MLL* aberrations, the concordance rate reaches almost 100% (Greaves, 2002) and retrospective studies have shown the clonality of the rearrangements (Gale *et al.*, 1997). According to Greaves and Wiemels (2003), the Knudson model, in addition to the twin concordance data, indicates that for every child with a particular translocation-positive leukemia, there has to be a greater number of healthy individuals that harbor the same translocation in a silent pre-leukemic clone. Similar studies of umbilical cord blood samples have shown that the frequencies of *ETV6/RUNX1* and *RUNX1/ETO*, for example, are 100 times higher in neonates than in pediatric leukemia patients (Mori *et al.*, 2002). These rearrangements may occur in a high proportion of developing fetuses, but without the production of functional chimeric proteins; alternatively, they could originate through inappropriate cellular conditions (Kim-Rouille *et al.*, 1999).

Specific breaks involving the *MLL* gene can be induced by a variety of stimuli associated with cellular stress or apoptosis, such as serum starvation or treatment with cytosine arabinoside (Stanulla *et al.*, 1997; Betti *et al.*, 2001; Vaughan *et al.*, 2005). The activation of some components of the apoptotic process under these conditions has been demonstrated (Alam *et al.*, 1999), and cells can recover the normal phenotype in the absence of phagocytic signals (Reddien *et al.*, 2001). Based on these considerations, it seems plausible that *MLL* rearrangements in normal individuals could result from exposure to genotoxic agents. The involvement of epipodophylotoxins in anomalies of this gene in therapy-related leukemias, and the evidence that neonatal leukemia originates *in utero*, have led to the hypothesis that maternal exposure to topoisomerase II inhibitors during pregnancy could be associated with an increased risk of leukemia (Ross, 2000). Synthetic and natural flavonoids bind to topoisomerase II to form a cleavable complex, despite the paradoxical finding that in some cases these compounds are anticarcinogenic (Greaves, 1997). Strick *et al.* (2000) demonstrated that natural flavonols such as quercetin and fisetin induced the same level of breaks at 11q23 as did etoposide, whereas luteolin and genistein were two-fold less effective than this drug and, in some cases, their combination had a cumulative effect in inducing *MLL* cleavage.

Epidemiological studies have shown a significant association between infant leukemias and maternal exposure to various chemicals (Shu *et al.*, 1996, 1999; Schuz *et al.*, 2000; Ma *et al.*, 2002; Mucci *et al.*, 2004). In the specific case of infant leukemia with *MLL* gene fusions, a case-control study identified significant variations in the ingestion of herbal medicines, drugs (*e.g.*, Dipyrone), and insecticides (Alexander *et al.*, 2001). A similar study that focused on maternal diet concluded that the ingestion of fruits and vegetables during pregnancy usually diminished the general risk of leukemia, although in the case of AML *MLL*(+) exposure to certain natural topoisomerase II inhibitors appeared to increase the risk of disease (Spector *et al.*, 2005).

According to Wiemels *et al.* (1999), the exposure of mothers and fetuses to dietary, medicinal and environmental substances that interact with topoisomerase II can be orders of magnitude lower in terms of dose level than for drugs used in chemotherapy. However, in some cases, these compounds are as biologically active as the topoisomerase II inhibitors used to treat cancer. The most abundant natural sources of topoisomerase inhibitors in a normal diet are fruits, vegetables and grains, which are rich in isoflavonoids. The antioxidant effect of these substances has been widely demonstrated (Prior, 2003), although epidemiological studies have shown that a high ingestion of isoflavonoids does not mean a reduced risk for all types of cancer (Hertog *et al.*, 1994). In Asian countries, for example, the ingestion of isoflavonoids can reach 28 mg/day (Fukutake *et al.*, 1996; Nakamura *et al.*, 2000). The plasma concentration of these substances after ingestion is relatively high (Franke *et al.*, 1998; Watanabe *et al.*, 1998) and can persist for two days. This finding suggests that the repeated inclusion of certain foods in the diet may ensure elevated plasma levels of these compounds (Hollman *et al.*, 1997; de Vries *et al.*, 1998).

Wiemels *et al.* (1999) also suggested that inter-individual variation in drug metabolism by phase I and phase II detoxifying enzymes could play an important role in modulating the response to low doses of topoisomerase II inhibitors. Thus, for example, the frequency of *NQ01* (NAD(P)H: quinone oxido-reductase) low-activity alleles is 2.5 times lower in patients with *AF4/MLL* fusions than in the normal population. Similarly, polymorphisms in *CYP3A4* (which converts epipodophylotoxins into catechol metabolites) have been associated with an increased risk of leukemia (Felix *et al.*, 1999). Uncontrolled exposure to certain substances and their metabolites can also contribute to gene fusions. Thus, hybrid genes that are present at low frequencies in peripheral blood of normal individuals tend to be more common in exposed populations, as in the case of the *TCR*β/γ hybrid gene in agricultural workers exposed to pesticides (Lipkowitz *et al.*,1992) and the translocation t(14;18) in smokers (Bell *et al.*, 1995).

Our study group consisted of healthy non-smokers with no previous history of drug treatment or chronic use of medicines. However, the presence of *MLL* fusions in peripheral blood lymphocytes of these individuals may have been related to previous exposures to substances from a variety of sources. Since lymphocytes circulate continuously they are considered to be more vulnerable to chemical or physical agents than other cell types (Tucker and Preston, 1996).

Tumor-associated translocations in peripheral lymphocytes may be transitory since sequential blood samples were not always positive for gene fusions, as shown for the *BCR/ABL* hybrid gene (Biernaux *et al.*, 1995). Other authors have suggested that such rearrangements may be expressed in hematopoietic cells that have entered the apoptotic pathway and have already lost their relevance (Bose *et al.*, 1998). On the other hand, whereas the genetic regulation mediated by *MLL* is important during hematopoietic differentiation, the expression of this gene (or of the fusion products) may be irrelevant in mature cells.

This study is the first to report the presence of *MLL* fusion genes at a genomic level in peripheral blood lymphocytes of healthy adults. To date, all screenings of leukemia-associated rearrangements have been based on RT-PCR. The *AF4/MLL* fusion transcripts were initially described in normal individuals (Uckum *et al.*, 1998) but subsequent studies failed to detect any transcription of this rearrangement (Kim-Rouille *et al.*, 1999; Trka *et al.*, 1999). In agreement with the latter studies, our results show that blood cells do not express detectable levels of *AF4/MLL* transcripts and there is no *a fortiori* synthesis of the chimeric protein. In addition, sequencing of the PCR products, which provides breakpoint information, showed that in most cases the translocations were not *in frame*, thus strengthening the hypothesis that they may be tolerated for years without adverse consequences.

The biological significance of fusion genes and their respective chimeras in differentiated cells is still uncertain, and an important question remains about their oncogenic potential in healthy individuals. Nevertheless, the high proportion of *MLL* rearrangements in normal individuals suggests that 11q23 anomalies may possibly result from exposure to endogenous or exogenous DNA-damaging agents. In practical terms, the high susceptibility of the *MLL* gene to chemically-induced damage suggests that monitoring the aberrations associated with this gene in peripheral lymphocytes may be a sensitive assay for assessing genomic instability in individuals exposed to genotoxic stress.

## Figures and Tables

**Table 1:** *MLL* fusions detected by inverse PCR in peripheral blood lymphocytes from healthy individuals.

Control	Clone	Translocation detected	e-value*
Co 5	46	t(4;11)	3e^-99^; 8e^-50^
Co 8	47	t(4;11)(q21;q23)	2e^-14^; 6e^-42^
Co 11	48	t(9;11)	9e^-60^; 2e^-17^
	49	t(9;11)	1e^-74^; 1e^-15^
Co 18	51	t(4;11)(q21;q23)	0.008
Co 21	52	t(2;11)	8e^-04^; 8e^-04^
Co 28	53	t(9;11)	2e^-172^
Co 30	54	t(4;11)(q21;q23)	3e^-14^; 8e^-98^
Co 31	55	t(4;11)(q21;q23)	3e^-67^;4e^-131^
	55-2	t(9;11)	0.003; 7e^-146^
Co 33	56	t(4;11)(q21;q23)	3e^-58^
Co 37	57	t(4;11)(q21;q23)	6e^-13^; 1e^-161^
Co 39	59	t(1;11)	5e^-37^; 1e^-114^
Co 41	60	t(11;19)(q23;p13)	6e^-46^; 9e^-42^
	60-2	t(4;11)(q21;q23)	2e^-21^; 5e^-41^
Co 43	61	t(4;11)(q21;q23)	6e^-23^; 4e^-58^
Co 44	62	t(4;11)(q21;q23)	0.046
Co 49	63	t(4;11)(q21;q23)	5e^-38^; 2e^-145^
Co 50	64	t(4;11)(q21;q23)	3e^-57^
Co 53	66	t(4;11)(q21;q23)	1e^-09^
Co 54	67	t(4;11)(q21;q23)	1e^-12^; 2e^-14^
	68	t(4;11)(q21;q23)	2e^-16^; 7e^-35^
Co 58	70	t(4;11)(q21;q23)	6e^-15^; 5e^-77^
Co 61	72	t(4;11)(q21;q23)	2e^-58^
	73	t(9;11)	2e^-04^
Co 63	74	t(4;11)(q21;q23)	7e^-48^
Co 70	75	t(11;12)	5e^-37^;2e^-54^
Co 71	115	t(4;11)(q21;q23)	4e^-52^;1e^-24^
Co 72	116	t(4;11)(q21;q23)	8e^-18^
Co 84	117	t(4;11)(q21;q23)	5e^-63^
Co 97	118	t(9;11)	1e^-137^; 8e^-31^
Co 98	119	t(4;11)(q21;q23)	0.057
Co 99	120	t(11;12)	3e^-08^; 7e^-99^
Co 113	121	t(9;11)	2e^-13^
Co 114	122	t(4;11)(q21;q23)	2e^-57^; 2e^-36^

(*) value obtained by BLASTn analysis.

**Table 2:** Frequencies of *MLL* rearrangements and extra signals in peripheral blood lymphocytes from healthy individuals analyzed by FISH.

Controls	Rearrangements per 100 cells	Extra signals per 100 cells	Controls	Rearrangements per 100 cells	Extra signals per 100 cells
Co-1	0.00	0.30	Co-33	0.00	0.20
Co-2	0.10	0.20	Co-34	0.10	0.30
Co-3	0.10	0.30	Co-35	0.10	0.30
Co-4	0.00	0.20	Co-36	0.00	0.00
Co-5	0.10	0.30	Co-37	0.10	0.00
Co-9	0.09	0.59	Co-38	0.00	0.20
Co-10	0.00	0.79	Co-39	0.20	0.10
Co-11	0.00	0.09	Co-40	0.10	0.10
Co-12	0.00	0.09	Co-41	0.00	0.00
Co-13	0.00	0.49	Co-42	0.10	0.20
Co-14	0.00	0.00	Co-43	0.00	0.00
Co-15	0.00	0.00	Co-44	0.00	0.10
Co-16	0.00	0.49	Co-45	0.00	0.00
Co-17	0.00	0.09	Co-46	0.00	0.40
Co-18	0.30	0.30	Co-48	0.00	0.00
Co-20	0.00	0.39	Co-50	0.00	0.00
Co-21	0.00	0.00	Co-51	0.00	0.00
Co-22	0.00	0.00	Co-52	0.00	0.00
Co-24	0.00	0.00	Co-55	0.00	0.02
Co-26	0.00	0.09	Co-57	0.00	0.10
Co-27	0.00	0.00	Co-59	0.00	0.20
Co-28	0.09	0.19	Co-63	0.10	0.00
Co-30	0.20	0.60	Co-64	0.00	0.20
Co-31	0.00	0.20	Co-65	0.00	0.00
Co-32	0.00	0.60			
